# Supplementary material for: The AppNL-G-F mouse retina is a site for preclinical Alzheimer’s disease diagnosis and research
Source: Acta Neuropathol Commun. 2021 Jan 6;9:6. doi: 10.1186/s40478-020-01102-5 (PMC7788955; doi:10.1186/s40478-020-01102-5)
Supplement: Supplementary file 1 — Additional file 1: Supplementary figures [file 40478_2020_1102_MOESM1_ESM.pdf]

Supplementary information

The *App*$^{NL\text{-}G\text{-}F}$ mouse retina is a site for preclinical Alzheimer's disease diagnosis and research

Marjan Vandenabeele[1,2], Lien Veys[1,2], Sophie Lemmens[3], Xavier Hadoux[4], Géraldine Gelders[1,2], Luca Masin[1,2], Lutgarde Serneels[2,5], Jan Theunis[6], Takashi Saito[7,8], Takaomi C. Saido[7], Murali Jayapala[9], Patrick De Boever[6,10], Bart De Strooper[2,5], Ingeborg Stalmans[3], Peter van Wijngaarden[4], Lieve Moons[1,2], Lies De Groef[1,2]*

[1]KU Leuven, Neural Circuit Development and Regeneration Research Group, Department of Biology, Leuven, Belgium

[2]Leuven Brain Institute, Leuven, Belgium

[3]University Hospitals Leuven, Department of Ophthalmology, Leuven, Belgium; KU Leuven, Research Group Ophthalmology, Department of Neurosciences, Leuven, Belgium

[4]Centre for Eye Research Australia, Royal Victorian Eye and Ear Hospital, East Melbourne, Australia; Ophthalmology, Department of Surgery, University of Melbourne, Parkville, Australia

[5]VIB Center for Brain & Disease Research, Leuven, Belgium; KU Leuven, Laboratory for the Research of Neurodegenerative Diseases, Department of Neurosciences, Leuven, Belgium

[6]VITO (Flemish Institute for Technological Research), Health Unit, Mol, Belgium

[7]Laboratory for Proteolytic Neuroscience, RIKEN Center for Brain Science, Saitama, Japan

[8]Department of Neurocognitive Science, Institute of Brain Science, Nagoya City University Graduate School of Medical Sciences, Japan

[9]Imec (Interuniversity Microelectronics Centre), Leuven, Belgium

[10] Hasselt University, Center of Environmental Sciences, Diepenbeek, Belgium; University of Antwerp, Department of Biology, Wilrijk, Belgium

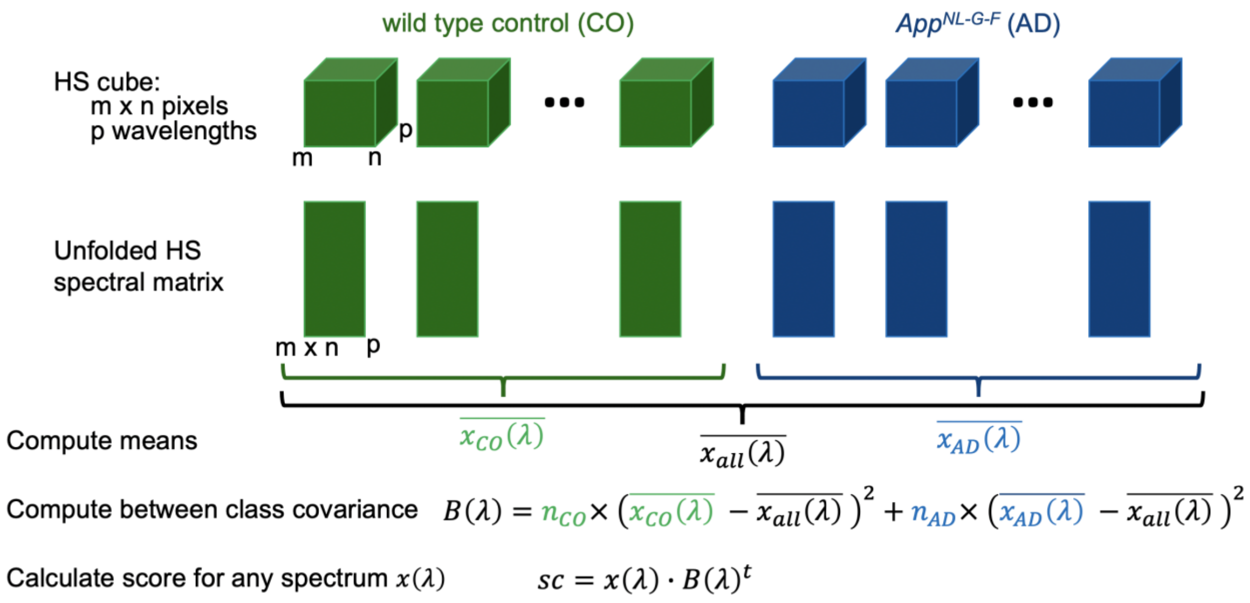

Supplementary figure 1. Schematic overview of the hyperspectral imaging data analysis. Spectral data from all mice were stacked in a matrix and a corresponding class label (AD or wild type control [CO]) was given for each entry. The between class covariance spectrum was calculated. This spectrum corresponds to the average spectral difference between AD and CO groups. The hyperspectral score for a given spectral pixel was obtained by calculating the dot product between its spectral data and the between class covariance spectrum. The average spectral score for a given mouse was calculated using all pixels of all the HS images collected for that retina.

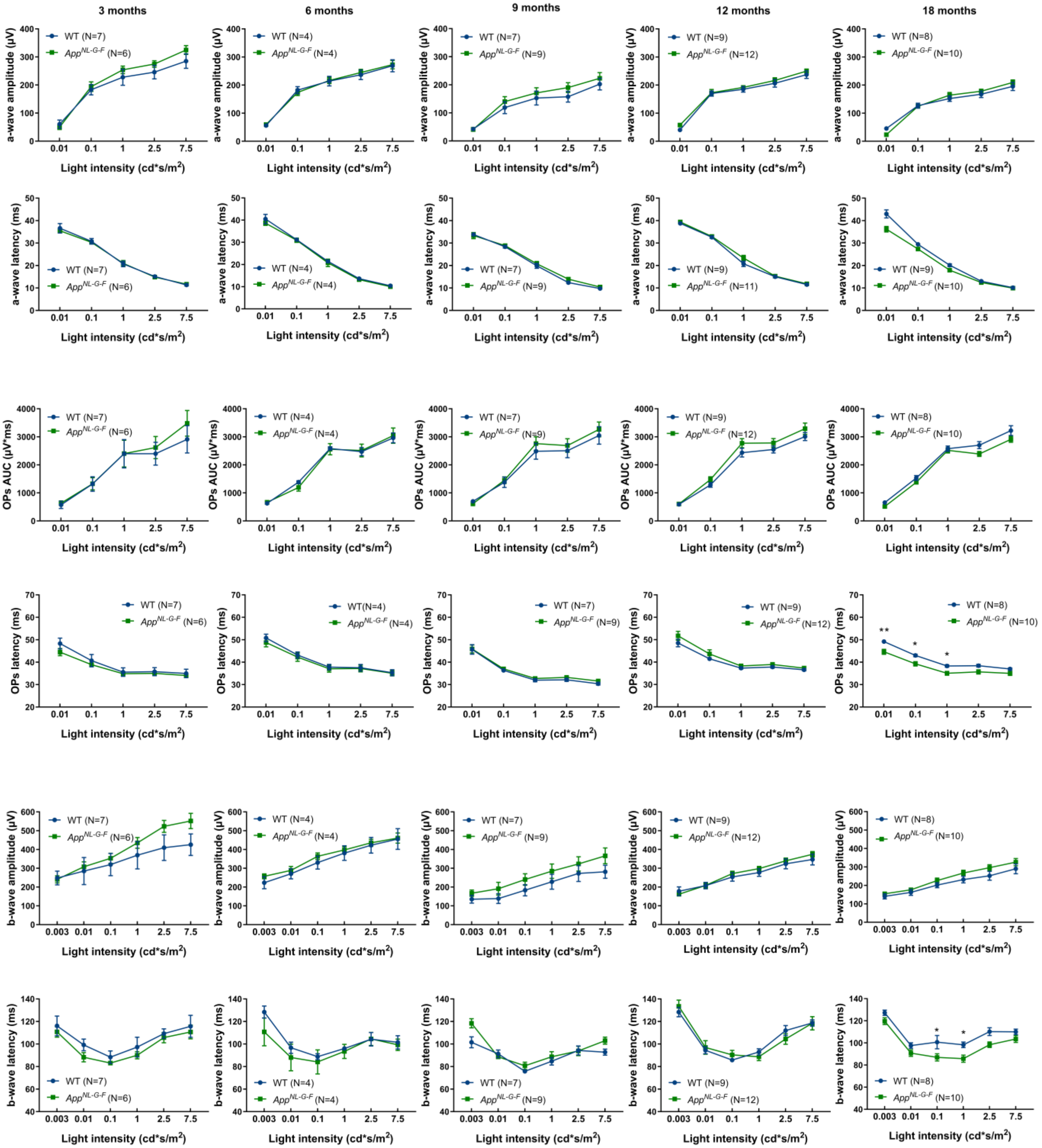
3 months
6 months
9 months
12 months
18 months
a-wave amplitude (µV)
WT (N=7)
App^NL-G-F (N=6)
WT (N=4)
App^NL-G-F (N=4)
WT (N=7)
App^NL-G-F (N=9)
WT (N=9)
App^NL-G-F (N=12)
WT (N=8)
App^NL-G-F (N=10)
a-wave latency (ms)
WT (N=7)
App^NL-G-F (N=6)
WT (N=4)
App^NL-G-F (N=4)
WT (N=7)
App^NL-G-F (N=9)
WT (N=9)
App^NL-G-F (N=11)
WT (N=9)
App^NL-G-F (N=10)
OPs AUC (µV*ms)
WT (N=7)
App^NL-G-F (N=6)
WT (N=4)
App^NL-G-F (N=4)
WT (N=7)
App^NL-G-F (N=9)
WT (N=9)
App^NL-G-F (N=12)
WT (N=8)
App^NL-G-F (N=10)
OPs latency (ms)
WT (N=7)
App^NL-G-F (N=6)
WT(N=4)
App^NL-G-F (N=4)
WT (N=7)
App^NL-G-F (N=9)
WT (N=9)
App^NL-G-F (N=12)
WT (N=8)
App^NL-G-F (N=10)
b-wave amplitude (µV)
WT (N=7)
App^NL-G-F (N=6)
WT (N=4)
App^NL-G-F (N=4)
WT (N=7)
App^NL-G-F (N=9)
WT (N=9)
App^NL-G-F (N=12)
WT (N=8)
App^NL-G-F (N=10)
b-wave latency (ms)
WT (N=7)
App^NL-G-F (N=6)
WT (N=4)
App^NL-G-F (N=4)
WT (N=7)
App^NL-G-F (N=9)
WT (N=9)
App^NL-G-F (N=12)
WT (N=8)
App^NL-G-F (N=10)
Light intensity (cd*s/m2)
0.003
0.01
0.1
1
2.5
7.5

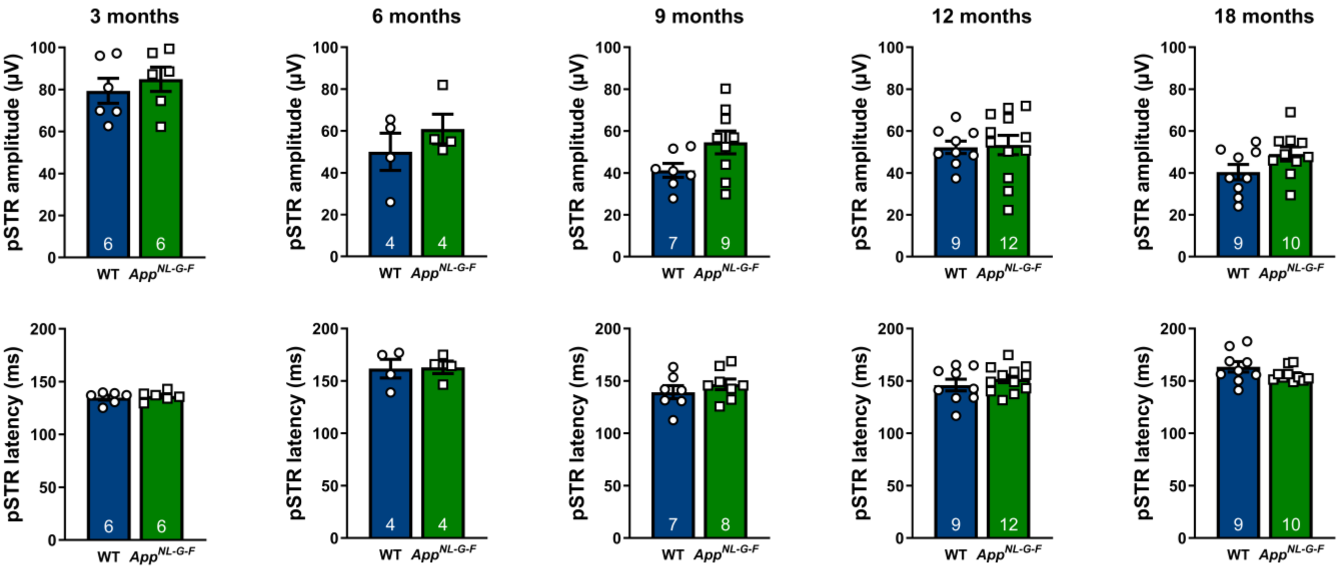

Supplementary figure 2. Electroretinogram measurements in *App^NL-G-F^* mice. Electroretinogram measurements were carried out in both *App^NL-G-F^* and WT mice at 3,6,9,12 and 18 months of age. Data are depicted as mean ± SEM; n=4-12 per group.
